# Utilization of Galerkin finite element strategy to investigate comparison performance among two hybrid nanofluid models

**DOI:** 10.1038/s41598-022-22571-9

**Published:** 2022-11-08

**Authors:** Muhammad Sohail, Umar Nazir, Samaira Naz, Abha Singh, Kanit Mukdasai, Mohamed R. Ali, Muhammad Jahangir Khan, Ahmed M. Galal

**Affiliations:** 1 Department of Mathematics, Khwaja Fareed University of Engineering and Information Technology, Rahim Yar Khan, 64200 Pakistan; 2 Department of Mathematics, Faculty of Science, Khon Kaen University, Khon Kaen, 40002 Thailand; 3 Department of Mathematics, Government College University, Faisalabad, Pakistan; 4 Department of Basic Science, College of Science and Theoretical Study, Dammam-Female Branch, Saudi Electronic University, Riyadh, Saudi Arabia; 5 Faculty of Engineering and Technology, Future University in Egypt, New Cario, 11835 Egypt; 6 Basic Engineering Science Department, Benha Faculty of Engineering, Benha University, Benha, Egypt; 7 Department of Advance Materials and Technologies, Faculty of Materials Engineering, Silesian University of Technology, 44-100 Gliwice, Poland; 8 Mechanical Engineering Department, College of Engineering, Prince Sattam Bin Abdulaziz University, Wadi Addawaser, 11991 Saudi Arabia; 9 Production Engineering and Mechanical Design Department, Faculty of Engineering, Mansoura University, P.O 35516, Mansoura, Egypt

**Keywords:** Mathematics and computing, Nanoscience and technology

## Abstract

The utilization of Fourier’s law of heat conduction provides the parabolic partial differential equation of thermal transport, which provides the information regarding thermal transport for the initial time, but during many practical applications, this theory is not applicable. Therefore, the utilization of modified heat flux model is to be used. This work discusses the utilization of non-Fourier heat flux model to investigate thermal performance of tri-hybrid nanoparticles mixture immersed in Carreau Yasuda material past over a Riga plate by using Hamilton Crosser and Yamada Ota models considering the variable thermos-physical characteristics. The phenomenon presenting the transport of momentum and energy are developed in the form of coupled partial differential equations, which are complex and then transformed into ordinary differential equations by using an appropriate transformation. The transformed equations have been tackled numerically via finite element scheme and the authenticity of obtained solution is shown with the help of comparative analysis of present results with those are available in open literature.

## Introduction

Industrial applications for hybrid nanofluids are still in the early stages of development. Hybrid nanofluids have only recently emerged as a new phenomenon, even though nanofluids have existed for decades. Hybrid nanofluids are expected to improve current application performance levels. A handful of hybrid nanofluid applications are currently being researched. They are expected to have the same density, heat capacity, and viscosity as their mono-component counterparts. The heat transfer coefficient can be significantly increased when two or more nanofluids are mixed. Researchers' interest in hybrid nanofluid applications has recently been piqued. Thermal storage, welding lubrication, transformer cooling, refrigeration, and biomedical and drug-reduction heat pipe cooling have many applications. The following are other potential uses: magnetic nanofluids have been used in various applications by researchers. Using a magnetic field can improve their ability to transfer heat.

It is possible to achieve thermal equilibrium with a wide variety of liquids. Fourier's law ignores the liquid's thermal relaxation characteristics when calculating heat transfer. The Fourier law makes it challenging to model heat transfer in fluids. These two scientists came up with a new heat conduction theory to solve this problem. Researchers came up with a new Fourier law for heat transfer in response to this new theory. Researchers frequently make use of these principles. Regardless of the outcome, our research is essential and must be completed. Reddy et al.^1^ estimated thermal enactment of hybrid nanoparticles in bio-magnetic pulsatile considering nanofluid in irregular channel. Xiu et al.^3^ discussed impacts of tri-hybrid nanoparticles in Reiner Philippoff liquid considering non-uniform Lorentz force past a stretching surface. They have adopted FEM to conduct numerical consequence and estimated comparison among hybrid nanoparticles and tri-hybrid nanoparticles. They have included that thermal enhancement for tri-hybrid nanofluid is better than thermal performance for hybrid nanoparticles. A study by Dogonchi and colleagues^4^ investigated the effect of nanoparticles on fluid heat transfer. They have used heat transfer theory to determine the thermal relaxation time. Al-Mdallal et al.^5^ visualized entropy optimization in pseudoplastic nano-polymer in occurrence of Lorentz force past a circular cylinder. Basha et al.^6^ utilized finite element method to obtain results of bio fluid associated with hybrid nanofluid in the presence of Lorentz force in stenosis artery. Reddy et al.^7^ performed role of entropy generation in peristaltic fluid considering nanofluid based on gold-blood in a microchannel. Basha and Sivaraj^8^ discussed results of entropy generation in Eyring–Powell fluid in the presence of biomedical applications in heated channel. In addition, it appears that numerous relevant works^9–12^ have been cited as well.

The heat transfer mechanisms are strikingly similar to those governing solute distribution in liquids. To incorporate the generalized Fourier heat transfer law into Fick's equations, scientists had to conduct prior research on the Fick law and the generalized Fourier heat transfer law. Fick's law of mass and heat transfer in Prandtl fluids is the focus of this study (non- Newtonian fluid). The current investigation will be better positioned if prior studies are reviewed. In the presence of nanoparticles, thermal transport is significantly accelerated. According to Haneef and colleagues^13^, the Cattaneo-Christov rheological fluid has heat and mass flux. Nawaz et al.^14^ studied the temperature-dependent coefficients of viscoelastic fluids using a theory other than the Fourier transform. The thermal act of a micro-polar fluid with monocity and hybridity was evaluated by Nawaz and his colleagues using a novel heat flux theory.

Recent years have seen a rise in interest in fluids that can be used in various industrial and domestic contexts. The list includes ink, nail polish, ketchup, and even wall paint. On the condiment bar, ketchup and whipped cream are included. Shear-thinning, pseudo-plastic, and plastic fluid are all terms that can be used interchangeably. As a result of the shear-thinning effect, fluids flow more easily under shear-thinning stresses. Oil paint, cream, and other mediums can benefit significantly from this feature. In a team led by Eberhard, The power law theory was used for the first time to calculate an effective shear rate. They went into the study assuming that the permeability would remain constant. Materials were subjected to shear thickening and thinning tests by Rosti and Takagi. A wide range of distinctive features was thus discovered. Gul et al.^15^ solved the thin-film power-law model for slip lifting and drainage. Sketches and various fluid velocity parameters were used to estimate the flow rate and coefficient of skin friction. The slip parameter was found to increase with a decrease in velocity. Hussein et al. investigated Brownian motion and thermophoresis in nanofluids in a vertical cylinder apparatus. Curvature calculations on the fluid and the model were used to determine the speed reductions. Abdelsalam and Sohail^16^ found that bioconvection affects the flow of nanofluids with varying viscosities over an elongated bidirectional surface. It was discovered that the motile density profile and the Peclet and Lewis indices were linked. Brownian motion and time-dependent thermophoresis can be used to study the thermal and concentration relaxation times of Sutterby flows. With the help of boundary layer theory and a suitable similarity transformation, they were able to turn the physical model into a coupled PDE system (PDEs). As a result of this update, the model can now be used to investigate a broader range of physical phenomena. After the ODEs had been converted, they were examined. The Prandtl number was used to gauge the temperature. The Schmidt number was increased by increasing the solution's concentration. In Chu and colleagues^17^, activation energy and chemical reactions significantly impact nanofluid flow. There was a decrease in fluid velocity when the Keller box scheme was implemented. Basha and Sivaraj^18^ evaluated features of entropy generation inserting ${Fe}_{3}{O}_{4}$-blood nanofluid in porous surface. In the case of pseudo-plastic drainage and lifting, the relationship between velocity decrease and Stokes number established by Alam et al.^19^ can be used to solve the problem. The pseudo-plastic model with variable viscosity showed flow. This paragraph necessitates citations. A perturbation technique was used to increase the magnetic parameter value and the velocity to solve the boundary value problem. New parameters have also been added to the studies conducted in^20–22,29–31^ and references therein.

## Physical aspects of flow model

Two dimensional model regarding rheology of Carreau Yasuda martial is developed and flowing assumptions are observed as

**Figure 1:**
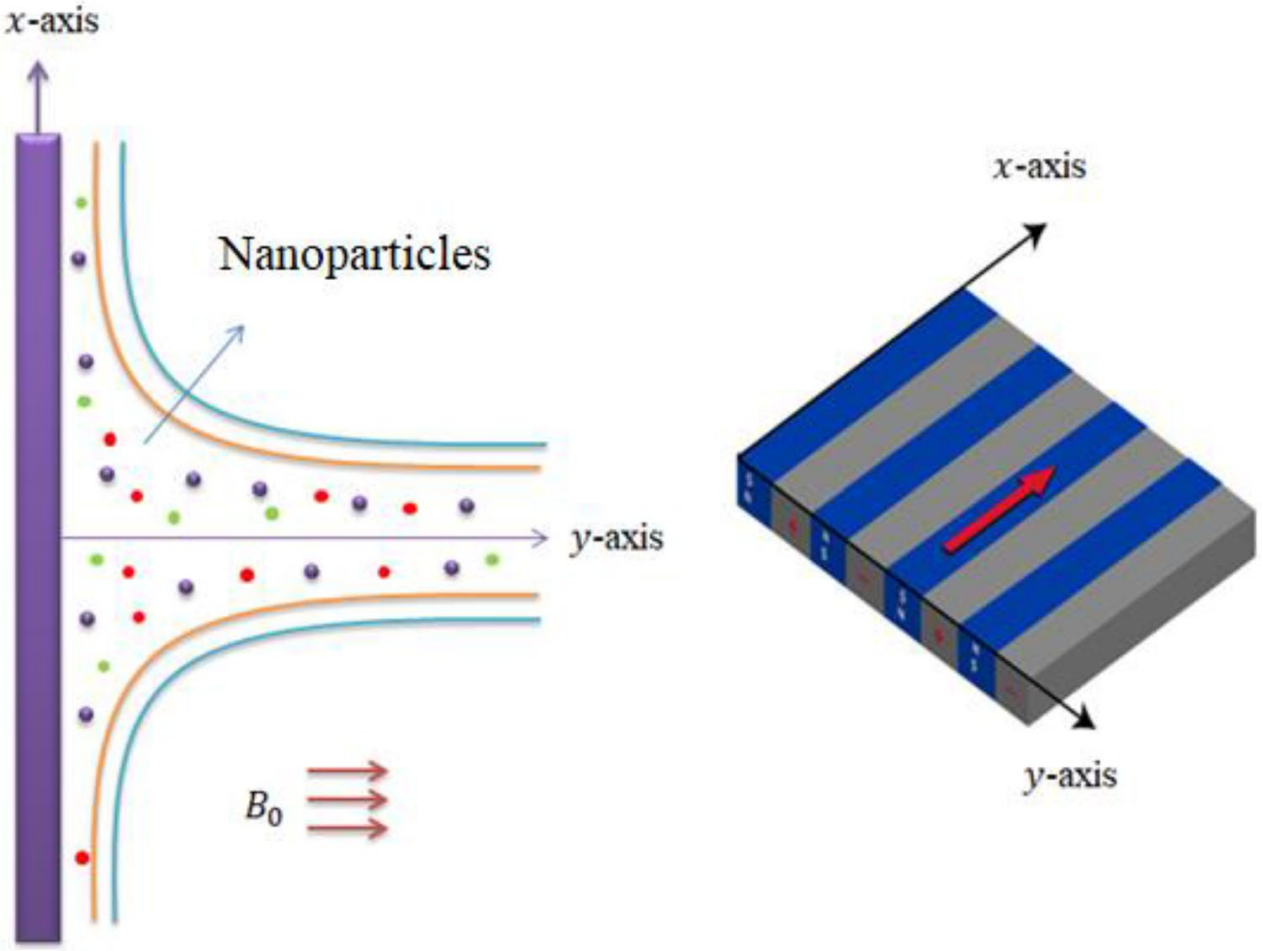
Geometry and coordinates system.

- Vertical Riga plate is considered;
- Two dimensional and incompressible flow are assumed;
- Heat generation and variable thermal conductivity are adopted;
- The suspension of ($Ti{O}_{2}/Si{O}_{2}$) in ethylene glycol is inserted;
- Lorentz force and bouncy forces are addressed;
- Two kinds of nanomaterial in EG (ethylene glycol) are imposed;
- Non-Fourier’s law is utilized;
- Hamilton Crosser and Yamada Ota models are imposed;
- Variable fluidic properties are addressed;
- The graphical representations of geometry are mentioned by Fig. 1.

Figure 1 shows a vertical surface and Riga plate. It is mentioned that y-axis is considered as horizontal and x-direction is assumed as a vertical direction. The constant magnetic field is inserted along y-direction whereas Riga plate is considered under electromagnetic force. Momentum and thermal boundary layers are generated. The motion of tri-hybrid nanoparticles is induced using wall velocity $\left({u}_{w}\right).$ The desired PDEs^23,24^ are obtained as

1 $$ \frac{{\partial V_{1} }}{\partial x} + \frac{{\partial V_{2} }}{\partial y} = 0, $$

2 $$ \begin{aligned} &V_{1} \frac{{\partial V_{1} }}{\partial x} + V_{2} \frac{{\partial V_{1} }}{\partial y} = \frac{{G\left( {\beta_{2} } \right)_{hybrid} \left( {T - T_{\infty } } \right)}}{{\rho_{hybrid} }} + \frac{{M_{0} \pi j_{0} }}{{8\rho_{hybrid} }}exp\left( { - \frac{\pi }{a}y} \right) \hfill \\ & \quad + \frac{\partial }{\partial y}\left[ {\mu_{hybrid}^{\theta } \left\{ {1 + {\Gamma }^{d} \left( {\left( {\frac{{\partial V_{1} }}{\partial y}} \right)^{d} } \right)^{{\left( {\frac{n - 1}{d}} \right)}} } \right\}\frac{{\partial V_{1} }}{\partial y}} \right], \hfill \\ \end{aligned} $$

3 $$ \begin{gathered} V_{1} \frac{\partial T}{{\partial x}} + V_{2} \frac{\partial T}{{\partial y}} + \gamma_{1} \left[ {\begin{array}{*{20}c} {V_{1}^{2} \frac{{\partial^{2} T}}{{\partial x^{2} }} + V_{2}^{2} \frac{{\partial^{2} T}}{{\partial y^{2} }} + 2V_{1} V_{2} \frac{{\dot{\partial }T}}{\partial x\partial y} + \left( {V_{1} \frac{{\partial V_{1} }}{\partial x} + V_{2} \frac{{\partial V_{1} }}{\partial x}} \right)\frac{\partial T}{{\partial x}}} \\ { + \left( {V_{1} \frac{{\partial V_{2} }}{\partial x} + V_{2} \frac{{\partial V_{2} }}{\partial x}} \right)\frac{\partial T}{{\partial y}} - \frac{{Q_{0} }}{{\left( {\rho C_{p} } \right)_{hnf} }}\left( {V_{1} \frac{\partial T}{{\partial x}} + V_{2} \frac{\partial T}{{\partial y}}} \right)} \\ \end{array} } \right] \hfill \\ = \frac{1}{{\left( {\rho C_{p} } \right)_{hybrid} }}\frac{\partial }{\partial y}\left( {k_{hybrid}^{T} \frac{\partial T}{{\partial y}}} \right) - \frac{{Q_{0} }}{{\left( {\rho C_{p} } \right)_{hybrid} }}\left( {T - T_{\infty } } \right). \hfill \\ \end{gathered} $$

BCs^23,24^ are

4 $${u}_{w}= cx={V}_{1}, {V}_{2}=0, T={T}_{w}, y :=0, {V}_{1}\to 0, T\to {T}_{\infty }:y\to \infty .$$

The desire transformations^23^ are delivered as

5 $$\eta =y{\left(\frac{{u}_{w}}{{x\nu }_{\infty }}\right)}^\frac{1}{2}, \frac{T-{T}_{\infty }}{{T}_{w}-{T}_{\infty }}=\uptheta , {V}_{1}=cx{F}{^{\prime}}, {V}_{2}=-\sqrt{{c\nu }_{f}}F.$$

Thermal condictivity in term of variable form^2^ which are

6 $${k}_{hybrid}^{t}={k}_{hybrid}\left[1+{\epsilon }_{1}\left(\frac{T-{T}_{\infty }}{{T}_{w}-{T}_{\infty }}\right)\right], \frac{1}{{\mu }_{hybrid}^{\theta }}=\frac{1+\gamma \left(T-{T}_{\infty }\right)}{{\mu }_{hybrid}}.$$

ODEs are achieved using Eq. (6) and obtained as

7 $$\frac{{\theta }_{\gamma }}{{\left({\theta }_{\gamma }-1\right)}^{2}}{\left\{1+\left(d+1\right)n{We}^{d}{F{^{\prime\prime}}}^{2}\right\}}^{\frac{n-1}{d}}{F}^{{^{\prime}}{{^\prime}}}{\theta }{^{\prime}}+\frac{{\nu }_{f}}{{\nu }_{hybrid}}\left(F{F}^{{^{\prime}}{{^\prime}}}-{F}{^{\prime}}{F}{^{\prime}}\right)+\frac{{\nu }_{f}}{{\nu }_{hybrid}}{\lambda }_{1}\uptheta +\frac{\omega exp}{{A}_{1}}\left(-\eta \beta \right)++\frac{{\theta }_{\gamma }}{{\theta }_{\gamma }-1}\left\{1+\left(d+1\right)n{We}^{d}{F{^{\prime}}{{^\prime}}}^{2}\right\}{\left\{1+\left(d+1\right)n{We}^{d}{F{^{\prime}}{{^\prime}}}^{2}\right\}}^{\frac{n-3}{d}}F{^{\prime}}{{^\prime}}{{^\prime}}=0,$$

8 $$ \begin{gathered} \left( {1 + \epsilon_{1} \theta } \right)\theta^{\prime\prime} + \epsilon_{1} \left( {\theta ^{\prime}} \right)^{2} - \beta_{a} \Pr \frac{{k_{f} \left( {\rho C_{p} } \right)_{hybrid} }}{{k_{hybrid} \left( {\rho C_{p} } \right)_{f} }}\left( {FF^{\prime}\theta^{\prime} + F^{2} \theta^{\prime\prime} + H_{t} F\theta^{\prime}} \right) + \frac{{k_{f} }}{{k_{hybrid} }}\Pr H_{t} \theta \hfill \\ + \frac{{k_{f} \left( {\rho C_{p} } \right)_{hybrid} }}{{k_{hybrid} \left( {\rho C_{p} } \right)_{f} }}\Pr F\theta^{\prime} = 0. \hfill \\ \end{gathered} $$

Using Eq. (6) in Eq. (5) and BCs are

9 $${F}{^{\prime}}\left(\infty \right)=0, \theta \left(0\right)=1, F\left(0\right)=0, {F}{^{\prime}}\left(0\right)=1, \theta \left(\infty \right)=0.$$

The correlations between two kinds of hybrid nanomaterial models^25^ are given below and the relationship between the physical quantities is mentioned in Table 1.

**Table 1:** Thermal properties^17^ of two kinds of nanofluid in EG (ethylene glycol).

	$K$	${C}_{p}$	$\rho$
${C}_{2}{H}_{6}{O}_{2}$	0.253	2430	1113.5
$Ti{O}_{2}$	8.4	692	4230
$Si{O}_{2}$	1.4013	$3.5\times {10}^{6}$	2270

10 $$\left.\begin{array}{c}{\rho }_{hybrid}=\left[\left(1-{\phi }_{2}\right)\left\{\left(1-{\phi }_{1}\right){\rho }_{f}+{\phi }_{1}{{\rho }_{s}}_{1}\right\}\right]+{\phi }_{2}{{\rho }_{s}}_{2}\\ {\left(\rho {C}_{p}\right)}_{hybrid}=\left[\left(1-{\phi }_{2}\right)\left\{\begin{array}{c}\left(1-{\phi }_{1}\right){\left(\rho {C}_{p}\right)}_{f}\\ +{\phi }_{1}{{\left(\rho {C}_{p}\right)}_{s}}_{1}\end{array}\right\}\right] {+\phi }_{1}{{\left(\rho {C}_{p}\right)}_{s}}_{2}\\ \left\{\frac{{{k}_{s}}_{1}+\left(m-1\right){k}_{f}-\left(m-1\right){\phi }_{1}\left({k}_{f}-{{k}_{s}}_{2}\right)}{{{k}_{s}}_{1}+\left(m-1\right){k}_{f}-{\phi }_{1}\left({{k}_{s}}_{2}-{k}_{f}\right)}\right\}=\frac{{k}_{bf}}{{k}_{f}}\end{array}\right\},$$

11 $$\left.\begin{array}{c}{\mu }_{hybrid}=\frac{{\left(1-{\phi }_{2}\right)}^{2.5}{\mu }_{f}}{{\left(1-{\phi }_{1}\right)}^{2.5}},\frac{{k}_{nf}}{{k}_{f}}=\left\{\frac{{k}_{s}+\left(m+1\right){k}_{f}-\left(m-1\right)\phi \left({k}_{f}-{k}_{s}\right)}{{k}_{s}+\left(m-1\right){k}_{f}+\phi \left({k}_{f}-{k}_{s}\right)}\right\}\\ \frac{{k}_{hybrid}}{{k}_{bf}}=\left\{\frac{{{k}_{s}}_{2}+\left(m-1\right){k}_{bf}-\left(1-m\right){\phi }_{2}\left({{k}_{s}}_{2}-{k}_{bf}\right)}{{{k}_{s}}_{2}+\left(m-1\right){k}_{bf}-{\phi }_{2}\left({k}_{bf}-{{k}_{s}}_{2}\right)}\right\}\\ \left\{\frac{{{k}_{s}}_{2}+\left(m-1\right){k}_{bf}-\left(1-m\right){\phi }_{2}\left({{k}_{s}}_{2}-{k}_{bf}\right)}{{{k}_{s}}_{2}+\left(m-1\right){k}_{bf}-{\phi }_{2}\left({k}_{bf}-{{k}_{s}}_{2}\right)}\right\}=\frac{{k}_{hybrid}}{{k}_{bf}}\end{array}\right\},$$

12 $$\left.\begin{array}{c}\frac{{k}_{hybrid}}{{k}_{bf}}=\left\{\frac{\frac{{{k}_{s}}_{2}}{{k}_{bf}}+\chi +\chi {\phi }_{2}\left(1-\frac{{{k}_{s}}_{2}}{{k}_{bf}}\right)}{\frac{{{k}_{s}}_{2}}{{k}_{bf}}+\chi +{\phi }_{2}\left(1-\frac{{{k}_{s}}_{2}}{{k}_{bf}}\right)}\right\}, \chi =2{{\phi }_{2}}^{0.2}\frac{L}{D} \, for\, cylindrical\,  particle\\ \chi =2{{\phi }_{2}}^{0.2}\,  for \, spherical\,  particle\end{array}\right\},$$

13 $$\left.\begin{array}{c}\frac{{k}_{bf}}{{k}_{f}}=\left\{\frac{\frac{{{k}_{s}}_{1}}{{k}_{f}}+\chi +\chi {\phi }_{1}\left(1-\frac{{{k}_{s}}_{1}}{{k}_{f}}\right)}{\frac{{{k}_{s}}_{1}}{{k}_{f}}+\chi +{\phi }_{1}\left(1-\frac{{{k}_{s}}_{1}}{{k}_{f}}\right)}\right\},\chi =2{{\phi }_{2}}^{0.5}\frac{L}{D} \, for \, cylindrical \, particle\\ \chi =2{{\phi }_{2}}^{0.5}\, for\,  spherical\,  particles\end{array}\right\}.$$

Parameters appeared in Eqs. (9)–(12) which are defined as

$${{\theta }_{\gamma }=\frac{1}{\gamma \left({T}_{w}-{T}_{\infty }\right)}, \beta }_{a}=c{\gamma }_{1}, We=\frac{\Gamma ax\sqrt{a}}{\sqrt{{\nu }_{f}}},Pr=\frac{{\left({C}_{p}\right)}_{f}{\mu }_{f}}{{k}_{f}}, {H}_{t}=\frac{{\pi M}_{0}{j}_{0}}{{\rho }_{f}8{u}_{w}a}, \beta ={\left(\frac{{{\pi }^{2}\nu }_{f}}{c{a}^{2}}\right)}^{1/2}.$$

Shear stress is defined as

14 $$Cf=\frac{{\tau }_{w}}{{\left({u}_{w}\right)}^{2}{\rho }_{f}},{\tau }_{w}={\mu }_{hybrid}{\left[\left(1+\left(\frac{n-1}{d}\right){\Gamma }^{d}{\left(\frac{\partial {V}_{1}}{\partial y}\right)}^{d}\frac{\partial {V}_{1}}{\partial y}\right)\right]}_{y=0}$$

Skin friction coefficient and temeprature gradient^23,24^ is delievered as

15 $${Re}^{1/2}Cf=-{\left(1-{\phi }_{1}\right)}^{-2.5}{\left(1-{\phi }_{2}\right)}^{-2.5}\left[1+\frac{n-1}{d}{\left(We{F}^{{^{\prime}}{{^\prime}}}\left(0\right)\right)}^{d}\right]{F}^{{^{\prime}}{{^\prime}}}\left(0\right),$$

16 $$Nu=\frac{xQ}{{\left(T-{T}_{\infty }\right)k}_{f}}=-\frac{{k}_{f}}{{{k}_{hybrid}Re}^{-\frac{1}{2}}}\left(1+{\epsilon }_{1}\right){\uptheta }{^{\prime}}\left(0\right),$$

## Numerical approach

**Figure 2:**
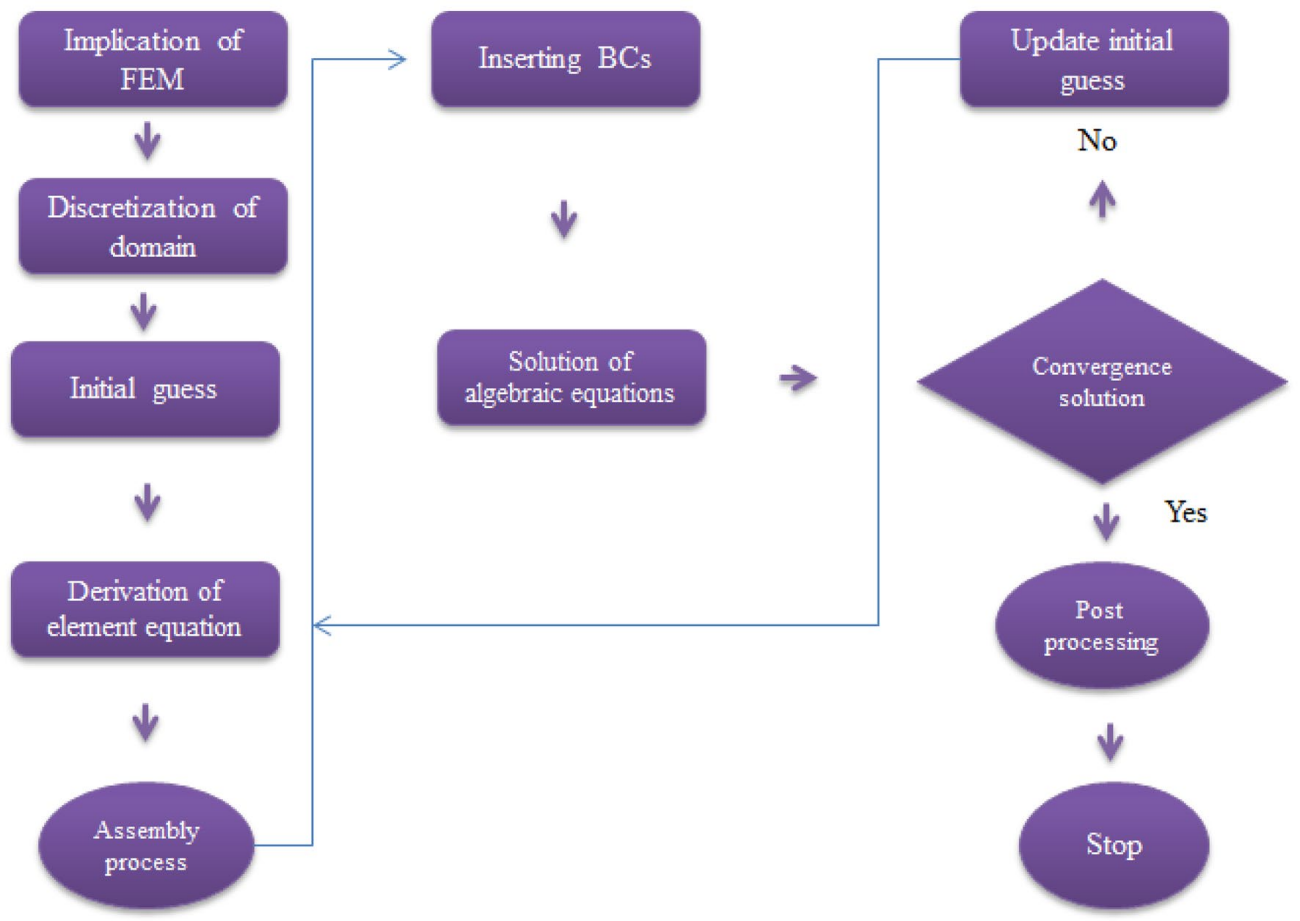
Flow chart of FEM.

Finite element apparoch is utlized to find numerical solution of resultant transformed ODEs (ordinary differential equations). Tables 2 and 3 are preapred to estimate grid size study and validation of problem. The proposed methodology is shown with the help of Fig. 2. Several advantages of finite element method are presecribed below.

- Complex geometric problems can be handled by FEM;
- Most of arising problems in applied science are resolved by FEM;
- It deals with different types of boundary conditions;
- Relatively required low investment, time and resources;
- It behaves significantly well in view of discretization of derivatives.

**Table 2:** Grid size study of concentration, teeprature and velocity for 300 elements when $We=3.0, d=1, {\lambda }_{1}=0.3, \beta =2.0, {\epsilon }_{1}=1.4, {\beta }_{a}=0.5, Pr=206, {H}_{t}=-2.0, Sc=3.0, {\phi }_{1}=0.004, {\phi }_{2}=0.0075, {\theta }_{\gamma }=-3.0.$

$e$	$F{^{\prime}}\left(\frac{{\eta }_{max}}{2}\right)$	$\theta \left(\frac{{\eta }_{max}}{2}\right)$
30	0.5721974488	0.3921033142
60	0.5460428484	0.3836649886
90	0.5373843928	0.3808296144
120	0.5330677499	0.5076407203
150	0.5304818169	0.5059713564
180	0.5287599374	0.5048579897
210	0.5275304862	0.3775697622
240	0.5266088495	0.5034653655
270	0.5258925734	0.5030028093
300	0.5253200716	0.5026336120

**Table 3:** Validation of study with already publisded works^27,28^ when $We=0, \beta =0, {\lambda }_{1}=0.$

$M$	Akbar et al.^27^	Bilal et al.^28^	Present study
0.0	− 1.0	− 1.0	− 1.0
0.5	− 1.11803	− 1.11800	− 1.11796
1.0	− 1.41419	− 1.41421	− 1.41421

### Residuals

The resiudulas^2^ of desired problem are

17 $${\int }_{{\eta }_{e}}^{{\eta }_{e+1}}{w}_{1}\left({F}{^{\prime}}-H\right)d\eta =0,$$

18 $${\int }_{{\eta }_{e}}^{{\eta }_{e+1}}{w}_{2}\left[\begin{array}{c}{H}^{{^{\prime}}{{^\prime}}}+\frac{\left(d+1\right)\left(n-1\right)}{d}{We}^{d}{H}^{{^{\prime}}{{^\prime}}{\left({H}{^{\prime}}\right)}^{d}}+\frac{{\nu }_{f}}{{\nu }_{hybrid}}\left(F{H}{^{\prime}}-{H}^{2}\right)\\ +\frac{{\nu }_{f}}{{\nu }_{hybrid}}{\lambda }_{1}\theta +\frac{\omega exp}{{A}_{1}}\left(-\eta \beta \right)\end{array}\right]d\eta =0,$$

19 $${\int }_{{\eta }_{e}}^{{\eta }_{e+1}}{w}_{3}\left[\begin{array}{c}\left(1+{\epsilon }_{1}\uptheta \right){\uptheta }^{{^{\prime}}{{^\prime}}}+{\epsilon }_{1}{\left({\uptheta }^{\mathrm{^{\prime}}}\right)}^{2}-{\beta }_{a}Pr\frac{{k}_{f}{\left(\rho {C}_{p}\right)}_{hybrid}}{{k}_{hybrid}{\left(\rho {C}_{p}\right)}_{f}}\left(FH{\uptheta }^{\mathrm{^{\prime}}}+{F}^{2}{\uptheta }^{\mathrm{^{\prime}}\mathrm{^{\prime}}}+{H}_{t}F{\uptheta }^{\mathrm{^{\prime}}}\right)\\ +\frac{{k}_{f}}{{k}_{hybrid}}Pr{H}_{t}\theta +\frac{{k}_{f}{\left(\rho {C}_{p}\right)}_{hybrid}}{{k}_{hybrid}{\left(\rho {C}_{p}\right)}_{f}}PrF{\uptheta }^{\mathrm{^{\prime}}}\end{array}\right]d\eta =0,$$

### Weak forms

The weak forms are developed using residual method. Shape function^2^ is

20 $${\psi }_{j}={\left(-1\right)}^{j-1}\left(\frac{-\eta +{\eta }_{j-1}}{-{\eta }_{j}+{\eta }_{j+1}}\right), i=1, 2.$$

### Approximations of Galerkin

Stiffness matrices^2^ are

21 $${{K}_{ij}^{14}=0,K}_{ij}^{11}={\int }_{{\eta }_{e}}^{{\eta }_{e+1}}\left(\frac{d{\psi }_{j}}{d\eta }{\psi }_{i}\right)d\eta , {K}_{ij}^{12}={\int }_{{\eta }_{e}}^{{\eta }_{e+1}}\left({\psi }_{j}{\psi }_{i}\right)d\eta , {B}_{i}^{1}=0,{K}_{ij}^{13}=0, {B}_{i}^{2}=0,$$

22 $${K}_{ij}^{22}={\int }_{{\eta }_{e}}^{{\eta }_{e+1}}\left[\begin{array}{c}-\frac{d{\psi }_{i}}{d\eta }\frac{d{\psi }_{j}}{d\eta }-\frac{\left(d+1\right)\left(n-1\right)}{d}{We}^{d}{\left(\overline{H }\right)}^{d}\frac{d{\psi }_{i}}{d\eta }\frac{d{\psi }_{j}}{d\eta }+\frac{{\nu }_{f}}{{\nu }_{hybrid}}\left(\overline{F}{\psi  }_{i}\frac{d{\psi }_{j}}{d\eta }\right)\\ -\frac{{\nu }_{f}}{{\nu }_{hybrid}}\overline{H}{\psi  }_{i}{\psi }_{j}\end{array}\right]d\eta ,{B}_{i}^{2}=0,$$

23 $${K}_{ij}^{23}=\left[\frac{{\nu }_{f}}{{\nu }_{hybrid}}{\lambda }_{1}{\psi }_{i}{\psi }_{j}\right]d\eta ,{K}_{ij}^{24}=\left[\frac{{\nu }_{f}}{{\nu }_{hybrid}}{\lambda }_{1}{\psi }_{i}{\psi }_{j}\right]d\eta , {K}_{ij}^{31}=0, {K}_{ij}^{32}=0, {K}_{ij}^{33}=0.$$

24 $${K}_{ij}^{33}={\int }_{{\eta }_{e}}^{{\eta }_{e+1}}\left[\begin{array}{c}\left(1+{\epsilon }_{1}\overline{\theta }\right)\frac{d{\psi }_{i}}{d\eta }\frac{d{\psi }_{j}}{d\eta }+{\epsilon }_{1}\left(\overline{{\theta  }{^{\prime}}}\right){\psi }_{i}\frac{d{\psi }_{j}}{d\eta }-{\beta }_{a}Pr\frac{{k}_{f}{\left(\rho {C}_{p}\right)}_{hybrid}}{{k}_{hybrid}{\left(\rho {C}_{p}\right)}_{f}}\left(\overline{F }\overline{H }\right){\psi }_{i}\frac{d{\psi }_{j}}{d\eta }\\ -{\beta }_{a}Pr\frac{{k}_{f}{\left(\rho {C}_{p}\right)}_{hybrid}}{{k}_{hybrid}{\left(\rho {C}_{p}\right)}_{f}}\left(\overline{{F }^{2}}\right)\frac{d{\psi }_{i}}{d\eta }\frac{d{\psi }_{j}}{d\eta }-{\beta }_{a}Pr\frac{{k}_{f}{\left(\rho {C}_{p}\right)}_{hybrid}}{{k}_{hybrid}{\left(\rho {C}_{p}\right)}_{f}}{H}_{t}\overline{F}{\psi  }_{i}\frac{d{\psi }_{j}}{d\eta }\\ +\frac{{k}_{f}}{{k}_{hybrid}}Pr{H}_{t}{\psi }_{i}{\psi }_{j}+\frac{{k}_{f}{\left(\rho {C}_{p}\right)}_{hybrid}}{{k}_{hybrid}{\left(\rho {C}_{p}\right)}_{f}}Pr\overline{F}{\psi  }_{i}\frac{d{\psi }_{j}}{d\eta }\end{array}\right]d\eta ,{B}_{i}^{1}=0.$$

### Computational tolerance

The computational tolerance is delivered as

25 $$\left|\frac{{\delta }_{i+1}-{\delta }_{i}}{{\delta }^{i}}\right|<{10}^{-5}.$$

### Estimation of error

Several methods are availbale t define error estimation. Residual based estimation^26^ is well known method for total energy norm which can be defined as

26 $$\Vert E\Vert = {\left(\sum_{i=1}^{n}{\Vert E\Vert }_{i}^{2}\right)}^{1/2}, {\Vert E\Vert }_{i}=\int \left(\nabla E\right){\left(\mathcal{L}E\right)}^{T}d\Omega .$$

where $E=f-\widehat{f}$ and $i$ reveals individual element. Energy norm can be delivered as

27 $${e}_{i}=\frac{\Vert E\Vert }{\Vert f\Vert }\times 100\%$$

## Results and its outcomes

The development of flow model regarding rheology of Carreau liquid over Riga heated plated is addressed in the presence of magnetic induction. Heat energy and heat transfer rate are visualized involving non-Fourier’s law inserting chemical reaction and heat absorption/heat generation. Three kinds of nanomaterial are inserted in EG. ODEs are simulated by FEM. Graphical results associated with heat energy against various parameters are mentioned below.

### Comparative outcomes regarding velocity field

**Figure 3:**
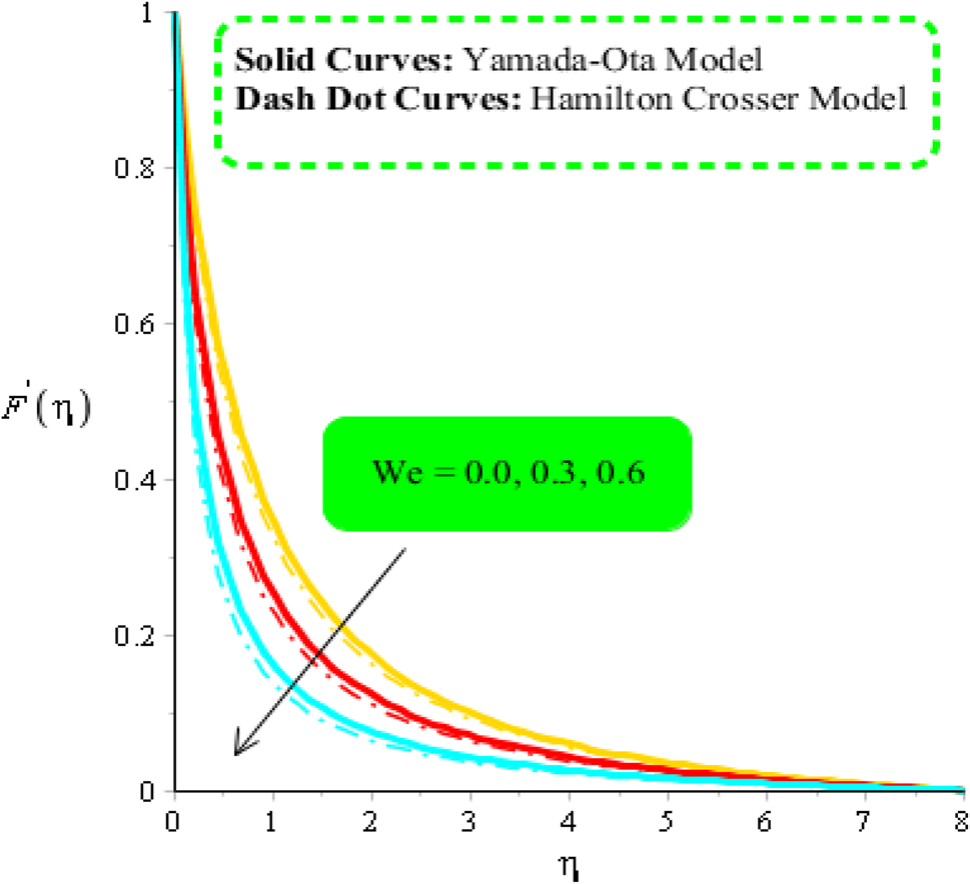
Comparison in velocity field against $We$ when $d=1, {\lambda }_{1}=0.3, \beta =2.0, {\epsilon }_{1}=1.4, {\beta }_{a}=0.5, Pr=206, {H}_{t}=-2.0, {\theta }_{\gamma }=-3.0, {\phi }_{1}=0.004, {\phi }_{2}=0.075.$

**Figure 4:**
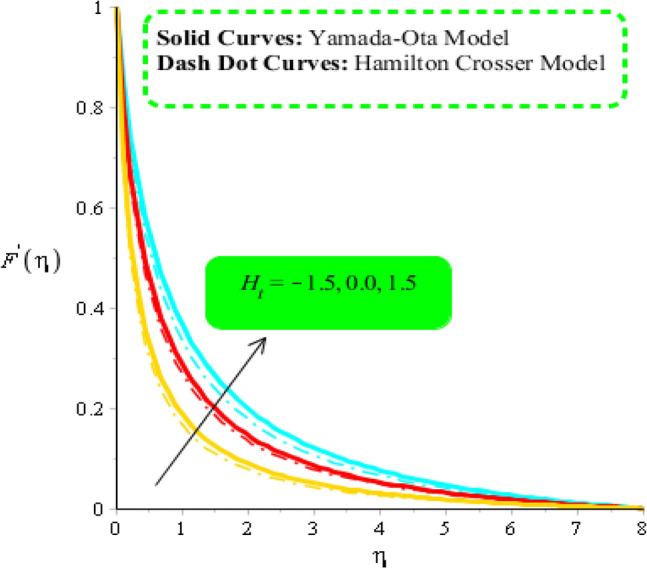
Comparison in velocity field against ${H}_{t}$ when $We=3.0, d=1, {\lambda }_{1}=0.3, \beta =2.0, {\epsilon }_{1}=1.4, {\beta }_{a}=0.5, Pr=206,{\theta }_{\gamma }=-2.0, {\phi }_{1}=0.004, {\phi }_{2}=0.075.$

**Figure 5:**
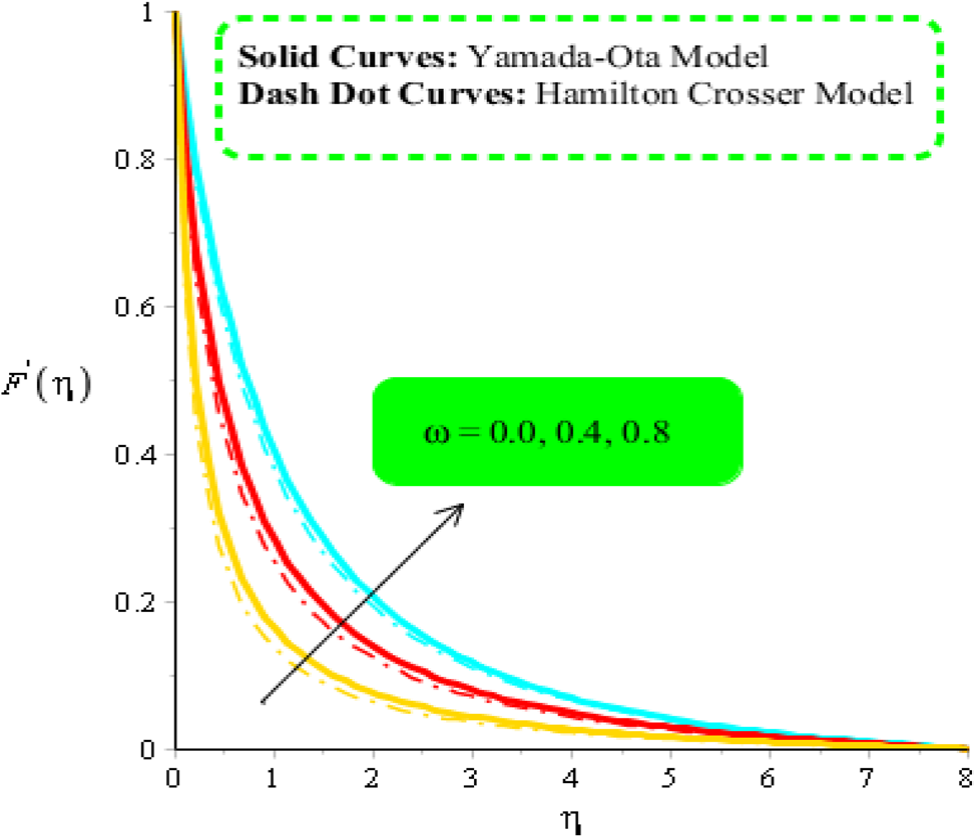
Comparison in velocity field against $\omega$ when $We=3.0, d=1, {\lambda }_{1}=0.3, \beta =2.0, {\epsilon }_{1}=1.4, {\beta }_{a}=0.5, Pr=206, {\theta }_{\gamma }=-3.0, {H}_{t}=-2.0, {\phi }_{1}=0.004, {\phi }_{2}=0.075.$

**Figure 6:**
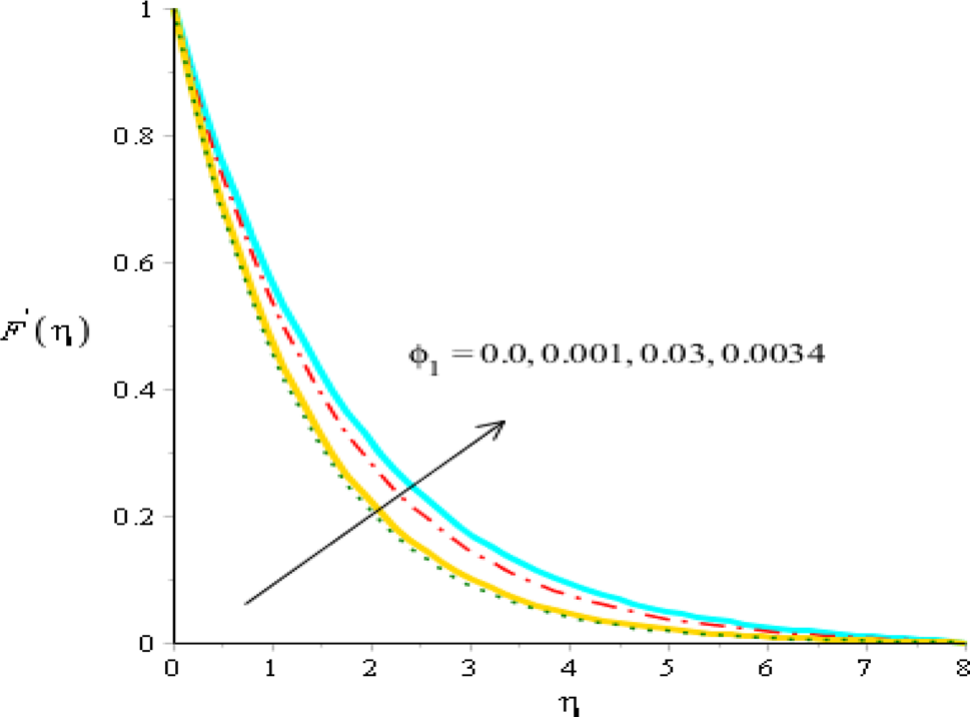
Behavior of velocity field against ${\phi }_{1}$ when $We=3.0, d=1, {\lambda }_{1}=0.3, \beta =2.0, {\epsilon }_{1}=1.4, {\beta }_{a}=0.5, Pr=206, {{\theta }_{\gamma }=-3.0, H}_{t}=-2.0, {\phi }_{2}=0.075, {\phi }_{1}=0.004.$

**Figure 7:**
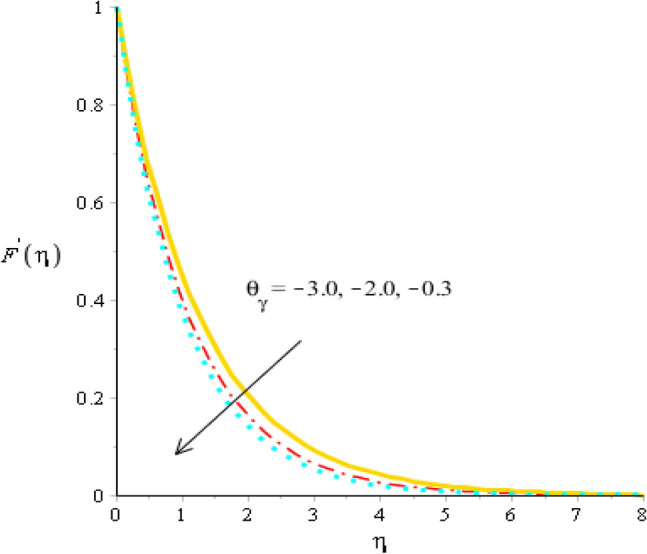
Behavior of velocity field against ${\theta }_{\gamma }$ when $We=2.0, d=1, {\lambda }_{1}=0.01, \beta =4.0, {\epsilon }_{1}=1.4, {\beta }_{a}=0.05, Pr=206, {H}_{t}=-3.0, {\phi }_{2}=0.075,{\phi }_{1}=0.004.$

Figures 3, 4 and 5 are plotted to measure comparative acceleration among two hybrid fluid models against change in several parameters. It is noticed that model-I is associated with Yamada-Ota hybrid model whereas model-II is considered by Hamilton Crosser hybrid model. Figure 3 is developed to notice relationship between velocity field and $We.$ It predicted that acceleration is decreased slowly when $We$ is enhanced. Physically, it is ratio between viscous force and frictional force. So, fluid becomes significantly viscous due to inverse proportional relation between $We$ and velocity distribution. It is noticed that appearance of $We$ is formulated using rheology of Carreau Yasuda in momentum equations. An inverse relation is visualized among flow and variation of $We.$ Therefore, it can be investigated that fluid becomes thinning when $We$ is enhanced. Further, flow for $We=0$ is higher than flow for $We \ne 0$. Flow is induced for case of hybrid nanofluid model-I is higher than flow for hybrid nanofluid model-II. An influence of ${H}_{t}$ on velocity distribution is carried out by Fig. 4. An implication heat source parameter accelerates maximum heat energy. In this, two types of behavior are addressed in term of heat generation and heat absorption. It is mentioned that heat generation process is occurred for ${H}_{t}>0$ and heat absorption process is occurred for ${H}_{t}<0$. Therefore, flow for ${H}_{t}>0$ is greater than flow for ${H}_{t}<0.$ Moreover, fluidic temperature is enhanced when heat generation process is occurred. Physically, an external heat source is utilized to control thickness of momentum boundary layers. MBLTs (momentum boundary layer thicknesses) for hybrid nanofluid-I is greater than MBLTs for the case of hybrid nanofluid-II. The role of $\omega$ on velocity distribution is carried out by Fig. 5. An acceleration into fluidic particles is augmented when $\omega$ is increased. The concept of $\omega$ is utilized during process of applying electromagnetic force in Riga plate. It can be noticed that appearance of $\omega$ is developed in last term of momentum equation $\frac{\omega exp}{{A}_{1}}\left(-\eta \beta \right).$ An electromagnetic force is utilized to enhancement flow when $\omega$ is increased. Figure 6 reveals effect of ${\phi }_{1}$ on velocity profile. It is numerically included that motion into particles is enhanced when ${\phi }_{1}$ is increased. The directly proportional impact for ${\phi }_{1}$ on flow is investigated in ethylene glycol. Behavior of ${\theta }_{\gamma }$ is carried out by Fig. 7. A decreasing trend is visualized on flow behavior when ${\theta }_{\gamma }$ is enhanced. It is studied that formulation of ${\theta }_{\gamma }$ is established when variable viscosity is addressed in present problem. Higher values of ${\theta }_{\gamma }$ are made declination into flow.

### Comparative outcomes regarding temperature field

**Figure 8:**
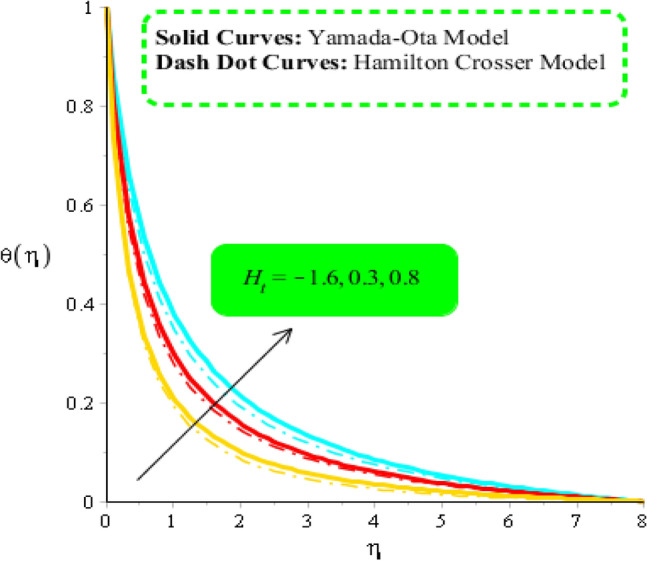
Comparison in temperature field against ${H}_{t}$ when $We=3.0, d=1, {\lambda }_{1}=0.3, \beta =2.0, {\epsilon }_{1}=1.4, {\beta }_{a}=0.5,{\theta }_{\gamma }=-2.0, Pr=206, {\phi }_{1}=0.004, {\phi }_{2}=0.075.$

**Figure 9:**
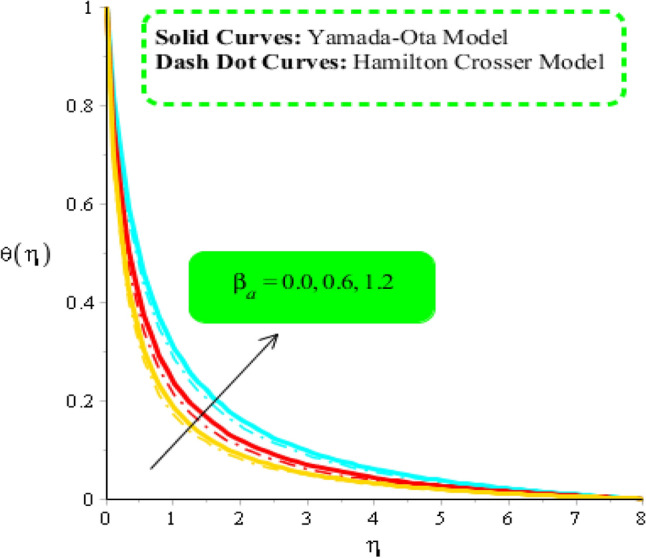
Comparison in temperature field against ${\beta }_{a}$ when $We=3.0, d=1, {\lambda }_{1}=0.3, \beta =2.0, {\epsilon }_{1}=1.4, {\beta }_{a}=0.5, Pr=206, {{\theta }_{\gamma }=-3.0, H}_{t}=-2.0, {\phi }_{1}=0.004, {\phi }_{2}=0.075.$

**Figure 10:**
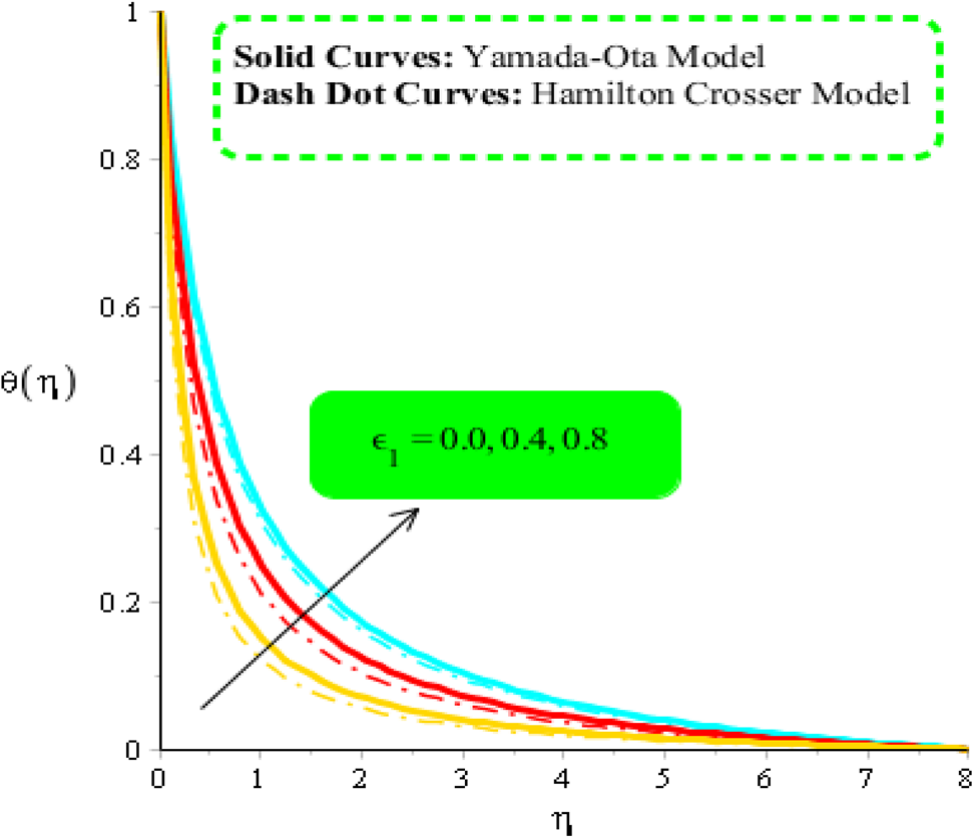
Comparison in temperature field against ${\epsilon }_{1}$ when $We=3.0, d=1, {\lambda }_{1}=0.3, \beta =2.0, {\beta }_{a}=0.5, Pr=206, {{\theta }_{\gamma }=-3.0, H}_{t}=-2.0, {\phi }_{1}=0.004, {\phi }_{2}=0.075.$

**Figure 11:**
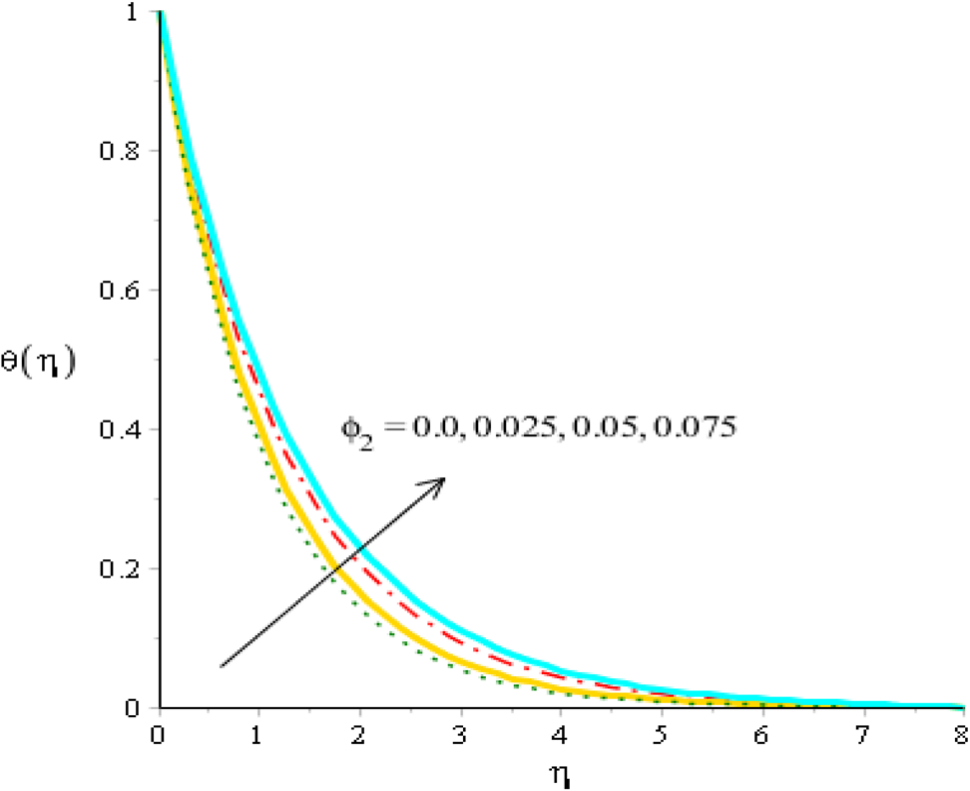
Comparison in temperature field against ${\phi }_{2}$ when $We=3.0, d=1, {\lambda }_{1}=0.3, \beta =2.0, {\beta }_{a}=0.5, Pr=206, {\theta }_{\gamma }=-3.0, {H}_{t}=-2.0, {\phi }_{1}=0.004.$

**Figure 12:**
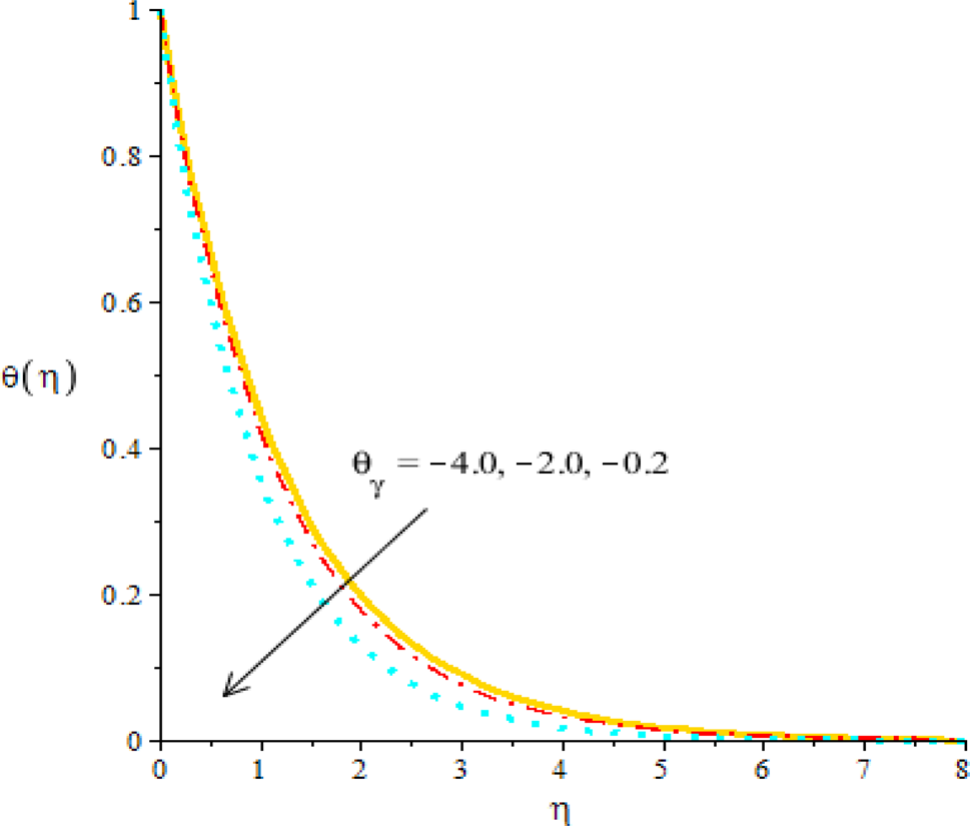
Behavior of temperature field against ${\theta }_{\gamma }$ when $We=4.0, d=0.3, {\lambda }_{1}=0.1, \beta =2.0, {\beta }_{a}=0.04, Pr=206, {H}_{t}=-4.0, {\phi }_{1}=0.004, {\phi }_{2}=0.075.$

Figures 8, 9 and 10 are developed to estimate variation in temperature field against heat source, ${\epsilon }_{1}$ and ${\beta }_{a}$. Figure 8 reveals increasing behavior of heat energy against change in ${H}_{t}.$ Heat energy was enhanced against increment in ${H}_{t}.$ This is happened when external heat source is utilized. It is noticed that heat generation process is occurred for ${H}_{t}>0$ and heat absorption process is occurred for ${H}_{t}<0$. Therefore, flow for ${H}_{t}>0$ is greater than flow for ${H}_{t}<0.$ Moreover, fluidic temperature is enhanced when heat generation process is occurred. Thermal performance for Yamada Ota model is greater than thermal performance for Hamilton Crosser model. Thermal layer thickness is also increasing function when ${H}_{t}$ is enhanced. Figure 9 captures an estimation of heat energy against variation in ${\beta }_{a}.$ It is investigated that ${\beta }_{a}$ is developed using concept of CCHFM (Cattaneo-Christov heat flux model) in energy and concentration equations. Time relaxation parameter restores maximum heat energy among fluidic particles. Therefore, heat energy is enhanced when ${\beta }_{a}$ is increased. The concept of ${\beta }_{a}$ is produced conspiring non-Fourier’s procedure in energy equation as well as in concentration equation. It is utilized to visualized thermal flux among wall and fluid. An enhancement into fluidic temperature is occurred because of direct proportional relation among thermal layers and ${\beta }_{a}.$ Fig. 10 reveals an impact of ${\epsilon }_{1}$ on temperature distribution. It is addressed that heat energy is increased against change in ${\epsilon }_{1}.$ Mathematically, ${\epsilon }_{2}$ has directly proportional relation versus mass diffusion rate. From Eq. (7), ${\epsilon }_{2}$ is existed in such function (function has domain of temperature). Mass diffusion rate is boosted when ${\epsilon }_{2}$ is enhanced. Mass diffusion for ${\epsilon }_{2}=0$ is less than for the case of ${\epsilon }_{2}\ne 0$. Basically, Therefore, heat energy is inclined. TBLT (thermal boundary layer thickness) for Yamada Ota model is higher than TBLT for the case Hamilton Crosser model. Figure 11 is plotted to measure heat energy versus impact of ${\phi }_{2}.$ It is visualized that heat energy is boosted when ${\phi }_{2}$ is increased. This is because ${\phi }_{2}$ is appeared due to occurrence of hybrid nanoparticles ($Ti{O}_{2}/Si{O}_{2}$) in base fluid named as ethylene glycol. Thermal energy can be boosted by adding an increment of ${\phi }_{2}$ into particles. Figure 12 reveals effect of ${\theta }_{\gamma }$ on temperature profile. Reduction into fluidic heat energy is investigated by considering higher values of ${\theta }_{\gamma }.$ It is happened due to appearance of variable viscosity.

### An estimation regarding wall stress and temperature gradient

Table 4 is prepared to measure consequences of $We, {H}_{t}$ and ${\epsilon }_{1}$ on wall stress and heat energy rate. It is estimated that divergent velocity and heat energy rate are declined versus the change in ${H}_{t}.$ But divergent velocity is enhanced versus the change in $We$. These outcomes are recorded in Table 4. Table 5 demonstrates impact of heat transfer rate against variation in $Pr, {\beta }_{a}$ and ${\lambda }_{1}.$ From Table 5, it is included that thermal performance of heat transfer rate is significantly decreased when $Pr, {\beta }_{a}$ and ${\lambda }_{1}$ are enhanced. The outcomes regarding heat transfer rate are recommended in Table 5.

**Table 4:** Simulations of divergent velocity (wall stress), Nusselt number and mass diffusion rate against ${\epsilon }_{1}, {H}_{t}$ and $We.$

Variation in parameters	${-Re}^{1/2}Cf$	${-Re}^{-\frac{1}{2}}NU$
***WE***		
0.0	0.04083083709	0.8718781018
0.4	0.04115641821	0.7608320013
0.8	0.04548199932	0.5334280014
$${{\varvec{H}}}_{{\varvec{t}}}$$		
− 1.5	0.05842070236	0.6865965216
0.0	0.02884959667	1.023554468
0.5	0.01075945969	1.268425453
$${{\varvec{\epsilon}}}_{1}$$		
0.0	0.02606740874	2.133026234
0.3	0.03568914202	0.687196814
0.5	0.1784160787	0.169312154

**Table 5:** Simulations of Nusselt number rate against $Pr, {\lambda }_{1}$ and ${\beta }_{a}$ when $We=3.0, d=1, {\lambda }_{1}=0.3, \beta =2.0, {\epsilon }_{1}=1.4, {\phi }_{1}=0.004, {\phi }_{2}=0.075, {H}_{t}=-3.0.$

Variation in parameters	${-Re}^{-\frac{1}{2}}Nu$
$${\varvec{P}}{\varvec{r}}$$	
203	0.37123950368
205	0.33066012203
206	0.31062012239
$${{\varvec{\lambda}}}_{1}$$	
0.0	0.96120133102
0.6	0.81663202181
0.9	0.76023217182
$${{\varvec{\beta}}}_{{\varvec{a}}}$$	
0.0	0.52322106912
2.0	0.42205522643
3.0	0.2245533610

## Main findings

The numerical investigation has been performed to discuss the contribution of nanoparticles for the thermal enhancement in Carreau Yasuda liquid past over a Riga plate in the presence of variable properties. The derived equations are tackled numerically and important findings are reported as

- Augmenting values of $We$ increase the dimensionless stress at boundary but depreciate the mass and heat transfer rates;
- Maximum performance of heat energy rate can be achieved with source of hybrid nanoparticles as applicable in coolants related to automobiles, dynamics of fuel, pharmaceutical processes, vehicle thermal adjustment, cooling process, microelectronics, temperature enhancement and temperature reduction;
- Comparative study have been performed to ensure the authenticity of solution;
- Convergence analysis has been shown through grid independent analysis and three hundred elements are taken to establish the convergence;
- The present problem related to electro-magneto-hydrodynamic has applicable in micro coolers, fluidic network flow, fluidic chromatography and thermal reactors.

## Data Availability

The datasets used and/or analysed during the current study available from the corresponding author on reasonable request.

## Abbreviations

$${V}_{1}, {V}_{2}$$: Velocity components
$$G$$: Gravitational acceleration
$$\rho $$: Fluid density
$$d$$: Fluid number
$${C}_{p}$$: Specific heat
$${T}_{\infty }$$: Ambient temperature
$$\Gamma $$: Fluid number
$$c$$: Stretching number
$${u}_{w}$$: Wall velocity
$${T}_{w}$$: Wall temperature
ODEs: Ordinary differential equation
$$hybrid$$: Hybrid nanofluid
$bf, f$: Base fluid and fluid
${H}_{t}$: Heat source number
$$Re$$: Reynolds number
$Q, {q}_{m}$: Wall heat flux and wall mass flux
${\beta }_{a}$: Time relaxation parameter
$$\infty $$: Infinity
$$F$$: Dimensionless velocity
$${s}_{1}, {s}_{2}$$: Solid particles
$${w}_{2}, {w}_{1}, {w}_{3}$$: Weight functions
$$\eta $$: Independent variable
$E, {\theta }_{\gamma }$: Energy and variable viscosity parameter
$$Si{O}_{2}$$: Silicon dioxide
$L$: Length of cylindrical particles
$$y,x$$: Space coordinates
${\beta }_{1}$: Coefficient of thermal and solute concentration
${M}_{0}, {J}_{0}$: Electromagnetic force components
$${Q}_{0}$$: Heat source
$$T$$: Fluid temperature
$$k$$: Thermal conductivity
$${K}_{M}$$: Chemical reaction
$$\eta $$: Independent parameter
${\nu }_{\infty }$: Kinematic viscosity from away of surface
${\epsilon }_{1}$: Thermal conductivity and mass diffusion coefficients
$$We$$: Wiesenberger number
$${\lambda }_{1}$$: Bouncy parameters
$$\theta $$: Dimensionless temperature
$${\phi }_{1}$$: Volume fractions
$$Pr$$: Prandtl number
$Cf$: Skin friction coefficient
$$Nu$$: Nusselt number
$$\mu $$: Kinematic viscosity
FEM: Finite element method
BCs: Boundary conditions
$\omega$: Electromagnetic magnetic force parameter
PDEs: Partial differential equations
$${\psi }_{j}$$: Shape function
$$Ti{O}_{2}$$: Titanium dioxide
$${C}_{2}{H}_{6}{O}_{2}$$: Ethylene glycol
$D$: Diameter of cylindrical particles
