# Author Correction: Utilization of Galerkin finite element strategy to investigate comparison performance among two hybrid nanofluid models

**DOI:** 10.1038/s41598-023-32048-y

**Published:** 2023-03-27

**Authors:** Muhammad Sohail, Umar Nazir, Samaira Naz, Abha Singh, Kanit Mukdasai, Mohamed R. Ali, Muhammad Jahangir Khan, Ahmed M. Gala

**Affiliations:** 1 Department of Mathematics, Khwaja Fareed University of Engineering and Information Technology, Rahim Yar Khan, 64200 Pakistan; 2 Department of Mathematics, Faculty of Science, Khon Kaen University, Khon Kaen, 40002 Thailand; 3 Department of Mathematics, Government College University, Faisalabad, Pakistan; 4 Department of Basic Science, College of Science and Theoretical Study, Dammam-Female Branch, Saudi Electronic University, Riyadh, Saudi Arabia; 5 Faculty of Engineering and Technology, Future University in Egypt, New Cairo, 11835 Egypt; 6 Basic Engineering Science Department, Benha Faculty of Engineering, Benha University, Benha, Egypt; 7 Department of Advance Materials and Technologies, Faculty of Materials Engineering, Silesian University of Technology, 44-100 Gliwice, Poland; 8 Mechanical Engineering Department, College of Engineering, Prince Sattam Bin Abdulaziz University, Wadi Addawaser, 11991 Saudi Arabia; 9 Production Engineering and Mechanical Design Department, Faculty of Engineering, Mansoura University, Mansoura, P.O 35516 Egypt

Correction to: *Scientific Reports* 10.1038/s41598-022-22571-9, published online 08 November 2022

The original version of this Article contained an error in Affiliation 5, which was incorrectly given as ‘Faculty of Engineering and Technology, Future University, Cario, Egypt’. The correct affiliation is listed below:

Faculty of Engineering and Technology, Future University in Egypt, New Cairo, 11835, Egypt

In addition, the original version of this Article omitted an affiliation for Mohamed R. Ali. The correct affiliations are listed below.

Faculty of Engineering and Technology, Future University in Egypt, New Cairo, 11835, Egypt

Basic Engineering Science Department, Benha Faculty of Engineering, Benha University, Benha, Egypt

The original Article has been corrected.

